# Device-measured sitting time and musculoskeletal pain in adults with normal glucose metabolism, prediabetes and type 2 diabetes–The Maastricht Study

**DOI:** 10.1371/journal.pone.0285276

**Published:** 2023-05-04

**Authors:** Francis Q. S. Dzakpasu, Annemarie Koster, Neville Owen, Bastiaan E. de Galan, Alison Carver, Christian J. Brakenridge, Annelies Boonen, Hans Bosma, Pieter C. Dagnelie, Simone J. P. M. Eussen, Parneet Sethi, Coen D. A. Stehouwer, Nicolaas C. Schaper, David W. Dunstan

**Affiliations:** 1 Mary MacKillop Institute for Health Research, Australian Catholic University, Melbourne, Victoria, Australia; 2 Physical Activity Laboratory, Baker Heart and Diabetes Institute, Melbourne, Victoria, Australia; 3 Department of Social Medicine, Maastricht University, Maastricht, the Netherlands; 4 CAPHRI Care and Public Health Research Institute, Maastricht University, Maastricht, the Netherlands; 5 Centre for Urban Transitions, Swinburne University of Technology, Melbourne, Victoria, Australia; 6 Department of Internal Medicine, Maastricht University Medical Centre, Maastricht, the Netherlands; 7 CARIM School for Cardiovascular Diseases, Maastricht University, Maastricht, the Netherlands; 8 Department of Internal Medicine, Radboud University Medical Centre, Nijmegen, the Netherlands; 9 National Centre for Healthy Ageing, Peninsula Clinical School, Monash University, Frankston, Victoria, Australia; 10 Active Life Lab, South-Eastern Finland University of Applied Sciences, Mikkeli, Finland; 11 Division of Rheumatology, Department of Internal Medicine, Maastricht University Medical Centre+, Maastricht, the Netherlands; 12 Department of Epidemiology, Maastricht University, Maastricht, the Netherlands; 13 Institute for Physical Activity and Nutrition (IPAN), School of Exercise and Nutrition Sciences, Deakin University, Geelong, Victoria, Australia; Maastricht University Faculty of Health, Medicine and Life Sciences: Maastricht Universitair Medisch Centrum+, NETHERLANDS

## Abstract

**Background:**

Detrimental associations of sedentary behaviour (time spent sitting) with musculoskeletal pain (MSP) conditions have been observed. However, findings on those with, or at risk of, type 2 diabetes (T2D) have not been reported. We examined the linear and non-linear associations of device-measured daily sitting time with MSP outcomes according to glucose metabolism status (GMS).

**Methods:**

Cross-sectional data from 2827 participants aged 40–75 years in the Maastricht Study (1728 with normal glucose metabolism (NGM); 441 with prediabetes; 658 with T2D), for whom valid data were available on activPAL-derived daily sitting time, MSP [neck, shoulder, low back, and knee pain], and GMS. Associations were examined by logistic regression analyses, adjusted serially for relevant confounders, including moderate-to-vigorous intensity physical activity (MVPA) and body mass index (BMI). Restricted cubic splines were used to further examine non-linear relationships.

**Results:**

The fully adjusted model (including BMI, MVPA, and history of cardiovascular disease) showed daily sitting time to be significantly associated with knee pain in the overall sample (OR = 1.07, 95%CI: 1.01–1.12) and in those with T2D (OR = 1.11, 95%CI: 1.00–1.22); this was not statistically significant in those with prediabetes (OR = 1.04, 95%CI: 0.91–1.18) or NGM (OR = 1.05, 95%CI: 0.98–1.13). There were no statistically significant associations between daily sitting time and neck, shoulder, or low back pain in any of the models. Furthermore, the non-linear relationships were statistically non-significant.

**Conclusion:**

Among middle-aged and older adults with T2D, daily sitting time was significantly associated with higher odds of knee pain, but not with neck, shoulder, or low back pain. No significant association was observed in those without T2D for neck, shoulder, low back, or knee pain. Future studies, preferably those utilising prospective designs, could examine additional attributes of daily sitting (e.g., sitting bouts and domain-specific sitting time) and the potential relationships of knee pain with mobility limitations.

## Introduction

Time spent sitting (sedentary behaviour) is associated with an increased risk of several adverse health outcomes, additional to the risks associated with insufficient levels of physical activity [1]. Specifically, there is evidence that higher volumes of daily sitting time are associated with all-cause mortality risk, along with increased risks of cardiovascular disease (CVD) and incident type 2 diabetes (T2D) [2–4].

Globally, the prevalence and burden of musculoskeletal pain (MSP)-related conditions are rising [5]. Also, there has been an increased focus on understanding the impact of MSP-related conditions as a comorbidity of T2D [6–8]. Some MSP conditions, for example, non-pyogenic tenosynovitis and stiff hand syndrome, are observed more common in those with diabetes [9]. Furthermore, neck, shoulder, low back, and knee osteoarthritic pain are well documented in those living with diabetes, particularly T2D [6–8, 10, 11]. T2D has also been linked with detrimental outcomes of some MSP conditions [7, 10]. Given that higher volumes of sitting time have been identified in those with T2D relative to those without T2D [12], sedentary behaviour could, in part, be a plausible contributor to MSP conditions in T2D [6, 13].

From a general population perspective, there is equivocal evidence on the relationships of sitting/sedentary time with MSP conditions in both cross-sectional and prospective studies [13–17]. High volumes of sitting time among some population cohorts, for instance, have been found to be associated with the increased risk of MSP conditions, such as low back pain, neck/shoulder pain, osteoarthritis, and general MSP [13, 14]. In contrast, studies have also documented either no evidence or inverse associations between sitting time and some MSP conditions [13, 15, 17]. In this context, the available evidence, most importantly those from population-based studies, has relied on self-report data on sitting time. There is limited evidence from studies using device-based measurement of sitting time, especially in large population-based samples; device-based studies have in the main utilised data from small subpopulations [13]. Also, it is unclear whether the relationships between sitting time and MSP conditions are linear or non-linear. Previous studies have mainly investigated the linear relationships of sitting time with MSP conditions [13, 15, 17], with a paucity of studies reporting on potential non-linear relationships. Further, the associations of sitting time with MSP conditions in adults according to glucose metabolism status (GMS), and especially on unique associations in those living with T2D, are unknown. Some evidence indicates the relationship of increased time spent in sedentary behaviour with changing pain severity in adults may be more pronounced in those with T2D [18].

We examined the cross-sectional associations of device-measured total daily sitting time with MSP outcomes–neck, shoulder, low back, and knee pain–in a large population-based sample of middle-aged and older adults and then separately in stratified subgroups of those with normal glucose metabolism (NGM), prediabetes, and T2D; we further examined potential non-linear relationships.

## Materials and methods

### Design and participants

The data were sourced from The Maastricht Study, an observational prospective population-based cohort study. The rationale and methodology have been described previously [19]. Briefly, the study focuses on the aetiology, pathophysiology, complications, and comorbidities of T2D and is characterized by an extensive phenotyping approach. Eligible for participation were all individuals aged between 40 and 75 years and living in the southern part of the Netherlands. Participants were recruited through mass media campaigns and from the municipal registries and the regional Diabetes Patient Registry via mailings. Recruitment was stratified according to known T2D status, with an oversampling of individuals with T2D, for reasons of efficiency [19].

For this cross-sectional study, 2827 participants from the full sample (N = 3451) who completed an initial survey between November 2010 and September 2013 –for whom there were data on musculoskeletal health, device-derived (activPAL) sitting time and physical activity, T2D status, and relevant covariates–were included in the analysis. The participants excluded were 126 without valid activPAL wear time, 24 with type 1 and other diabetes diagnoses, and 474 who had a missing variable of either exposure, outcome, or covariates. Little’s test of missing completely at random was performed to check whether the exposure and outcome variables were missing at random, as well as the covariate-dependent missingness and ensured the assumptions were met before running the complete-case analysis [20]. Participant examinations were performed within a time window of three months. The study has been approved by the institutional medical ethical committee (NL31329.068.10) and the Minister of Health, Welfare and Sports of the Netherlands (Permit 131088-105234-PG). All participants gave written informed consent.

### Measures

#### Outcomes–Musculoskeletal pain (MSP)

Data were based on a self-reported questionnaire on musculoskeletal health (validated in a Dutch sample) [19], which was adapted from the United States population-based validated Health Assessment Questionnaire used in the National Health and Nutrition Survey (NHANES) [21]. Participants were asked whether they had at least one instance of experiencing pain (yes/no) for the past one month in the following 11 body regions–neck, shoulder, elbow, wrist, hand, low back, hip, pelvis, knee, ankle, and foot; excluding pain as a result of trauma. They were also asked to indicate whether a physician had made a diagnosis for the pain. For this analysis, a pain episode for at least one day in the past one month in the neck, shoulder, low back, and knee was considered.

#### Exposure–Daily sitting time

The activPAL3 physical activity monitoring device (PAL Technologies, Glasgow, UK) was used to continuously measure participants’ sitting time, 24hr/day. The activPAL3 data collection, analytic processes, sitting time, and other physical activity time calculations have been described elsewhere [22]. Participants were instructed to wear the device for eight consecutive days without removing it until the final day. The first and the final days’ data were excluded because participants performed physical function tests on the first day while wearing the device, and the final day’s data were collected for less than 14-hours of waking time. Participants’ data were eligible for inclusion in the analysis if they had at least one valid day (more than 14-hours of waking data) device wear time. Time spent sitting during wake time on valid days derived from the activPAL device was used to calculate the mean daily sitting time in hours per day.

#### Covariates

Self-reported history of T2D and a standard 2-hour oral glucose tolerance test (OGTT) were used to ascertain participants’ GMS. Except for those with known T2D receiving insulin therapy who were captured by self-reported instrument, all other participants with unknown GMS underwent a standardised 7-time point OGTT after an overnight fast with 75g glucose ingestion, as described elsewhere [19]. World Health Organisation (WHO) criteria were used to categorise participants as NGM, prediabetes, and T2D [23]. Prediabetes was defined as impaired fasting glucose with fasting plasma glucose 6.1–6.9mmol/L and 2-hour postprandial plasma glucose less than 7.8mmol/L or impaired glucose tolerance with fasting plasma glucose less than 7.0mmol/L and 2-hour postprandial plasma glucose ≥7.8 and <11.1mmol/l. T2D was defined as fasting plasma glucose greater than 7.0mmol/L or 2-hour postprandial plasma glucose greater or equal to 11.1mmol/L [23], or known T2D and on glucose-lowering medications.

Moderate-to-vigorous intensity physical activity (MVPA) time was derived from the activPAL3 data as minutes with steps frequency more than 100 steps/min during waking hours as described elsewhere [24]. A general questionnaire was used to gather data for other covariates such as age, sex, level of education (categorised as low, medium, or high), and smoking status (never smoked, former smoker, current smoker). Participants’ dietary quality score was assessed with a validated Food Frequency Questionnaire [25] from which a Dutch Healthy Diet index (DHD-index) was derived, which is based on Dutch dietary guidelines [26]. Body mass index (BMI) was calculated from the physical examination data. Mobility limitation was based on participants’ self-report of any difficulty climbing one flight of stairs or walking 500 metres derived from the 36-item short-form health survey instrument. A self-reported history of CVD from the Rose questionnaire [27] was an additional confounding covariate.

### Statistical analyses

The characteristics of the study population were examined by GMS categories (NGM, prediabetes, and T2D). Continuous variables were calculated and summarised as means and standard deviations with differences between the NGM, prediabetes, and T2D subgroups examined using linear regression models by regressing the continuous variables as the outcome against the GMS and significant difference tested by using testparm (post-estimation command); whereas categorical variables were summarised as proportions (percentages) and a chi-square test used to compare the groups’ differences. To account for multiple-hypothesis testing in comparisons across the groups, a stringent p-value of < 0.01 was set as the significance level based on Bonferroni correction. Potential confounding variables were selected a priori based on prior literature. All statistical analyses were performed using STATA statistical software (StataCorp version 17), and the significance of associations in main analyses was considered at a p-value of ≤ 0.05 for the overall sample and those within the GMS groups.

**Fig 1:**
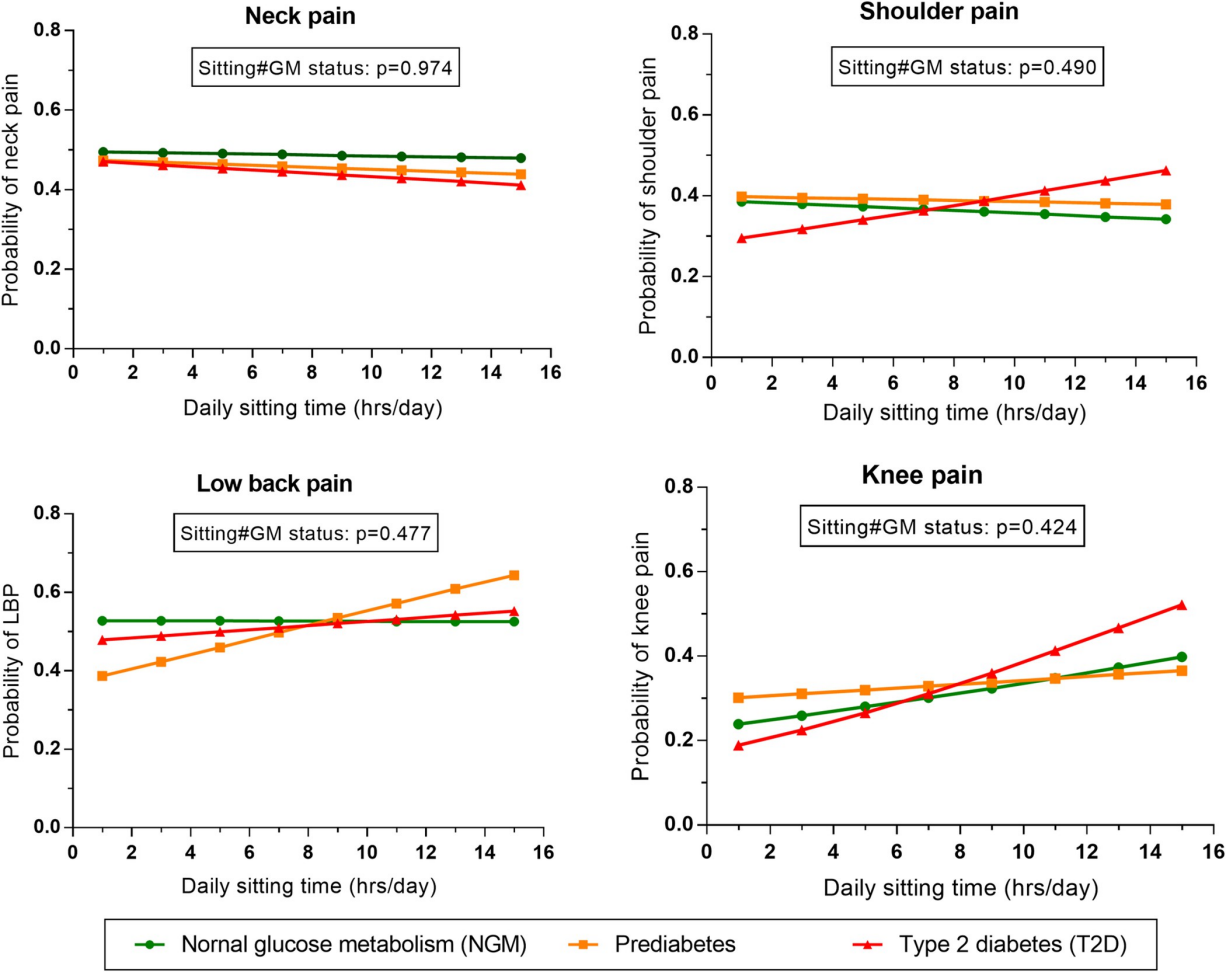
The predictive probability of the musculoskeletal pain outcomes with daily sitting time according to glucose metabolism status (GMS).

First, to examine the association of total daily sitting time with the MSP outcomes (neck, shoulder, low back, and knee pain), we used logistic regression modelling and statistically checked the a priori decision to stratify the analysis by GMS. Multiplicative interaction between daily sitting time and GMS was modelled for the MSP outcomes in the overall sample, adjusting for age and sex with the margins command used to estimate the predicted probability of the MSP outcome and marginal plot (line graphs) used to interpret the potential interactions (Fig 1). For the main analysis, progressively adjusted multiple logistic regressions were modelled, regressing each of the MSP outcomes (yes-MSP/no-MSP) as the dependent (outcome) variable and daily sitting time as the independent (exposure) variable for the overall sample and separately for NGM, prediabetes, and T2D. The first model (model A) was adjusted for age and sex.

**Fig 2:**
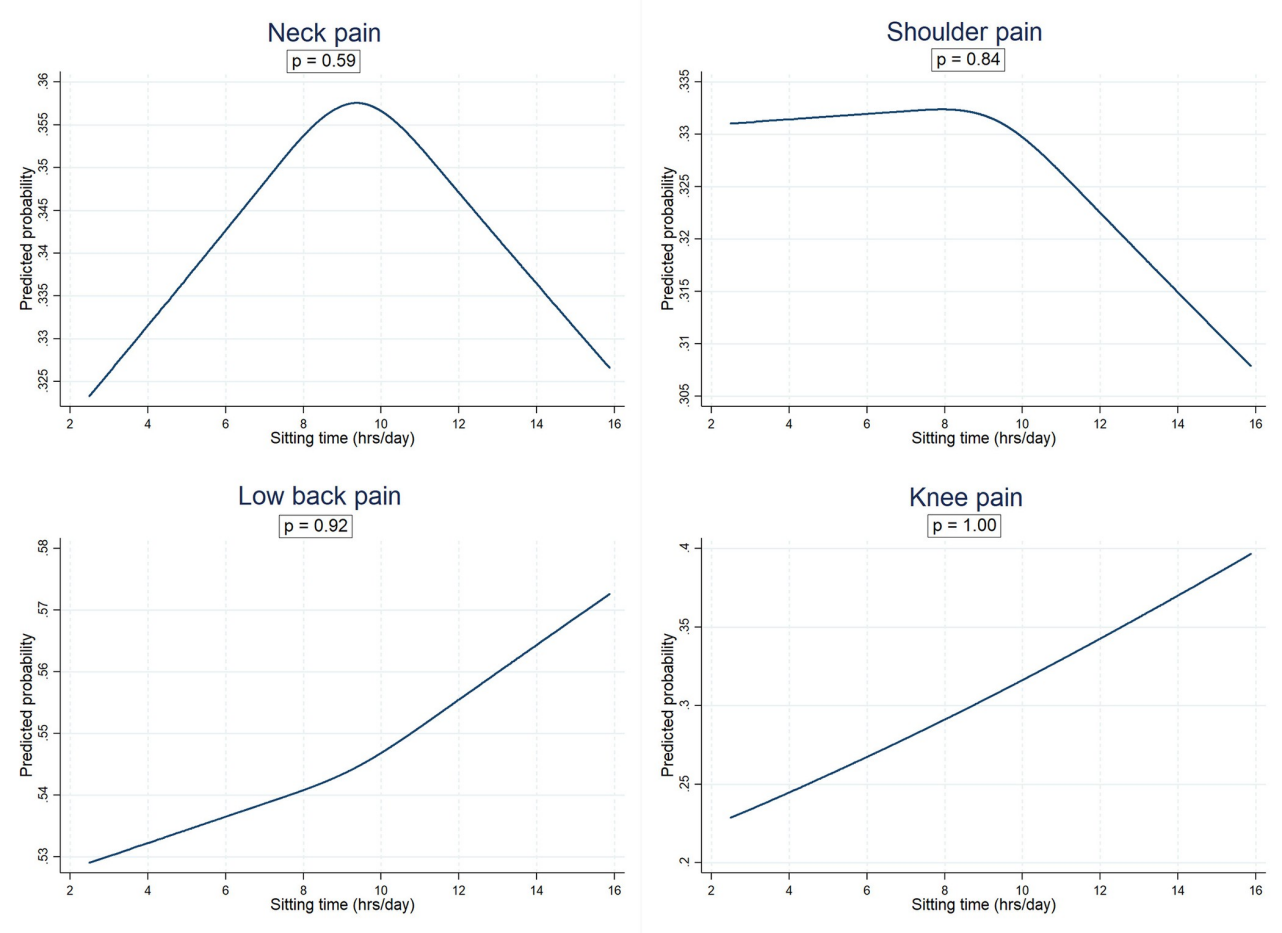
Non-linear relationships between daily sitting time and neck, shoulder, low back, and knee pain (overall sample with and without type 2 diabetes).

Second, the models were further adjusted for BMI and MVPA (Model B) to examine the attenuation effect on the direction of potential associations. Again, the fitted models were fully adjusted by adding some confounding variables, including socioeconomic variables (education level and employment status) and lifestyle variables (dietary quality score–DHD-index, and smoking status), as well as a history of CVD (Model C). Then, the robustness of the associations was examined by further adjusting for mobility limitation as a surrogate for other conditions that may predispose to excessive sedentary behaviour (Model D). Further, we examined the non-linear relationships of daily sitting time with the MSP outcomes using restricted cubic splines (RCS)–the most rigorous and flexible approach recommended for investigations of non-linear relationships [28, 29]. Three knots RCS (selected based on Akaike information criterion (AIC)–provided in the Supplementary file) were fitted (for the final fully adjusted models) and outputs were presented in line graphs (Fig 2 –for the overall sample and Supplementary S1 Fig in S1 File, as well as Supplementary S2a–S2d Fig in S1 File for the GMS subgroups–with scatter plots illustrations of distributions of the predicted probability of the MSP outcomes).

For sensitivity analyses, a multiplicative interaction of daily sitting time with sex was tested by modelling sitting time/sex interaction on the MSP outcomes. Also, we excluded all those with mobility limitations to check for the potential of reverse causality bias (25.9% of the total sample size) and re-ran the models.

The distributions of daily sitting with the MSP outcomes, as well as the linear and non-linear analytic models’ fitness checks, are provided in the Supplementary file.

## Results

Characteristics of the participants according to GMS are shown in Table 1. Those with T2D were relatively older, and on average, spent more hours sitting and fewer hours in MVPA compared to participants with pre-diabetes and NGM. Compared to those with NGM and prediabetes, those with T2D were more likely to be male, obese, have a history of CVD, and have mobility limitations.

**Table 1:** Characteristics of the study population.

Variables	Overall (N = 2,827)	NGM (N = 1,728)	Prediabetes (N = 441)	T2D (N = 658)	p-value
**Demographic**					
Age, mean (SD)	59.5 (8.6)	57.7 (8.5)	62.0 (8.1)	62.7 (7.9)	< 0.001
**Sex**					< 0.001
Female, n(%)	1,613 (57.1)	1,120 (64.8)	238 (54.0)	255 (38.7)	
BMI, mean. (SD), kg/m2	27.1 (4.6)	25.6 (3.8)	28.2 (4.4)	30.3 (5.0)	< 0.001
**Socioeconomic status**					
*Education level*					< 0.001
Low, n(%)	1,026 (36.3)	536 (31.0)	177 (40.1)	313 (47.6)	
Medium, n(%)	801 (28.3)	516 (29.9)	109 (24.7)	176 (26.8)	
High, n(%)	1,000 (35.4)	676 (39.1)	155 (35.2)	169 (25.7)	
*Employment status*					< 0.001
Unemployed, n(%)	1,579 (55.9)	848 (49.1)	275 (62.4)	456 (69.3)	
Employed, n(%)	1,186 (42.0)	842 (48.7)	155 (35.2)	189 (28.7)	
Other, n(%)	62 (2.2)	38 (2.2)	11 (2.5)	13 (2.0)	
**Lifestyle**					
Sitting time, mean(SD) hrs/day	9.2 (1.7)	9.0 (1.6)	9.2 (1.8)	9.9 (1.8)	< 0.001
MVPA, mean(SD) min/day	52.0 (25.4)	56.7 (25.2)	49.2 (23.3)	41.5 (23.8)	< 0.001
DHD-index score, mean(SD)	84.5 (15.1)	86.5 (14.7)	83.4 (14.8)	80.1 (15.1)	< 0.001
*Smoking status*					< 0.001
Never, n(%)	1,037 (36.7)	696 (40.3)	148 (33.6)	193 (29.3)	
Former, n(%)	1,433 (50.7)	825 (47.7)	245 (55.6)	363 (55.2)	
Current, n(%)	357 (12.6)	207 (12.0)	48 (10.9)	102 (15.5)	
**Medical history**					
*CVD history*					< 0.001
Yes, n(%)	493 (17.4)	240 (13.9)	69 (15.7)	184 (28.0)	
*Mobility limitation*					< 0.001
Yes, n(%)	732 (25.9)	318 (18.4)	136 (30.8)	278 (42.3)	

BMI: Body Mass Index | NGM: Normal Glucose Metabolism | T2D: Type 2 Diabetes | CVD: Cardiovascular Diseases | DHD-index: Dutch healthy diet index | Significance levels were set at p-value <0.01 to account for multiple-hypothesis testing across the groups.

For comparisons between the subgroups (NGM, prediabetes, and T2D)–continuous variables were examined by linear regression with post-estimation testparm; categorical variables were by chi-square test.

As shown in Table 2, the body region with the highest prevalence of MSP was low back pain (52.8%) and the least prevalent was knee pain (34.2%). The prevalence of knee pain was marginally non-significantly higher (p = 0.03 –with the significance level set at p < 0.01 to account for multiple testing) in the T2D group compared to the prediabetes and NGM groups, whereas the prevalence of neck pain was significantly higher (p < 0.001) in those with NGM than in the prediabetes and T2D groups. There were no statistical differences in the prevalence of shoulder or low back pain according to T2D status.

**Table 2:** Prevalence of musculoskeletal pain outcomes according to glucose metabolism status (GMS).

MSP outcomes	Overall (N = 2,827)	NGM (N = 1,728)	Prediabetes (N = 441)	T2D (N = 658)	p-value
Neck pain	1,328 (47.0)	870 (50.4)	194 (44.0)	264 (40.1)	< 0.001
Shoulder pain	1,062 (37.6)	653 (37.8)	165 (37.4)	244 (37.1)	0.948
Low back pain	1,494 (52.8)	919 (53.2)	235 (53.3)	340 (51.7)	0.788
Knee pain	966 (34.2)	562 (32.5)	152 (34.5)	252 (38.3)	0.029

MSP: Musculoskeletal pain | NGM: Normal Glucose Metabolism | T2D: Type 2 Diabetes | Significance levels were set at p-value <0.01 to account for multiple-hypothesis testing across the groups.

Numbers indicate the frequency of MSP; numbers in brackets are percentages.

The interaction term for daily sitting time and GMS was not statistically-significant for any of the MSP outcomes. However, the plotted predicted probability shows that there may be interactions for shoulder, low back, and knee pain as the lines for NGM, prediabetes, and T2D appear to cross each other as daily sitting time increases. This seems not to be the case for neck pain which has the lines for the groups being parallel to each other. Thus, there are indications that there may be variations in the associations of daily sitting with some of the MSP outcomes by GMS (illustrated in Fig 1). Specifically for knee pain, as the volume of daily sitting time increased, the predicted probability of knee pain non-significantly increased, which was more apparent in those with T2D than in those without–prediabetes and NGM (knee pain–p for interaction = 0.424). The interaction models are provided in Supplementary S1 Table in S1 File.

Table 3 presents the progressively-adjusted logistic regression findings of the linear relationships of daily sitting time with MSP outcomes for the overall sample, and separately for those with NGM, prediabetes, and T2D. A statistically significant association of daily sitting time with MSP outcomes was observed only for knee pain. In the fully adjusted model, including demographic and socioeconomic confounders, as well as BMI, MVPA, and history of CVD, daily sitting time was positively associated with increased odds of knee pain (OR = 1.07, 95%CI: 1.01–1.12). In analyses stratified by GMS, the relationship was significant only in those with T2D (OR = 1.11, 95%CI: 1.00–1.22), but not for those with prediabetes (OR = 1.04, 95%CI: 0.91–1.18) or those with NGM (OR = 1.05, 95%CI: 0.98–1.13). The associations remained statistically-significant in the overall sample (OR = 1.06, 95%CI: 1.01–1.12) and marginally significant in the T2D group (OR = 1.10, 95%CI: 1.00–1.22) after adjusting for mobility limitation in the robustness test. A further sensitivity check showed that there was no significant interaction with sex (results not shown). The significant associations were attenuated after excluding those with mobility limitations from the analysis to check for reverse causation, but there were few changes in the trend of the associations (results provided in Supplementary S2 Table in S1 File).

**Table 3:** Association of daily sitting time (hours/day) with musculoskeletal pain outcomes in the overall sample and separately in those with normal glucose metabolism, prediabetes, and type 2 diabetes.

MSP outcomes	N	Model A	Model B	Model C	Model D
		OR (95%CI)	OR (95%CI)	OR (95%CI)	OR (95%CI)
**Neck pain**					
Overall	2,827	0.98 (0.94–1.03)	0.99 (0.94–1.04)	1.00 (0.95–1.05)	1.00 (0.95–1.05)
NGM	1,728	0.99 (0.93–1.06)	1.00 (0.93–1.07)	1.01 (0.95–1.09)	1.02 (0.95–1.09)
Prediabetes	441	1.00 (0.89–1.11)	1.00 (0.89–1.13)	1.01 (0.89–1.14)	1.00 (0.89–1.14)
T2D	658	0.98 (0.89–1.07)	0.97 (0.88–1.08)	0.98 (0.89–1.08)	0.98 (0.88–1.08)
**Shoulder pain**					
Overall	2,827	1.01 (0.97–1.06)	0.99 (0.94–1.04)	0.99 (0.94–1.05)	0.99 (0.94–1.05)
NGM	1,728	0.99 (0.93–1.06)	0.98 (0.91–1.05)	0.99 (0.92–1.06)	0.98 (0.92–1.06)
Prediabetes	441	0.99 (0.89–1.11)	0.95 (0.84–1.08)	0.95 (0.84–1.07)	0.95 (0.84–1.07)
T2D	658	1.05 (0.96–1.15)	1.03 (0.93–1.13)	1.01 (0.92–1.12)	1.01 (0.91–1.12)
**Low back pain**					
Overall	2,827	1.02 (0.97–1.07)	1.01 (0.96–1.06)	1.02 (0.97–1.07)	1.01 (0.96–1.07)
NGM	1,728	0.99 (0.93–1.05)	0.99 (0.93–1.07)	1.00 (0.93–1.07)	0.99 (0.93–1.07)
Prediabetes	441	1.09 (0.97–1.21)	1.08 (0.96–1.22)	1.09 (0.97–1.23)	1.09 (0.97–1.24)
T2D	658	1.04 (0.95–1.13)	0.99 (0.90–1.09)	1.00 (0.90–1.10)	0.99 (0.90–1.09)
**Knee pain**					
Overall	2,827	**1.08 (1.02–1.13)**	**1.06 (1.01–1.12)**	**1.07 (1.01–1.12)**	**1.06 (1.01–1.12)**
NGM	1,728	1.06 (0.99–1.14)	1.05 (0.98–1.13)	1.05 (0.98–1.13)	1.05 (0.97–1.13)
Prediabetes	441	1.01 (0.90–1.13)	1.03 (0.91–1.17)	1.04 (0.91–1.18)	1.05 (0.92–1.19)
T2D	658	**1.11 (1.01–1.22)**	**1.10 (1.00–1.22)**	**1.11 (1.00–1.22)**	***1*.*10 (1*.*00–1*.*22)*** ^***$***^

Note: Complete-case analysis | The significant associations are shown in boldface (p ≤ 0.05) | ^*$*^ p = 0.055 | MSP: Musculoskeletal pain | N: Sample size | NGM: Normal Glucose Metabolism | T2D: Type 2 Diabetes | OR: Odds ratio | CI: Confidence Interval.

**Model A:** Adjusting for age and sex. **Model B:** Adjusting for covariates in Model A + BMI and MVPA. **Model C:** Adjusting for covariates in Model B + Education level, employment status, smoking status, DHD-index, and history of cardiovascular disease. **Model D:** Adjusting for covariates in Model C + mobility limitations.

There were no statistically significant associations in the overall sample or in the specific GMS groups between the daily sitting time and neck, shoulder, or low back pain in any of the models, as well as in the sensitivity tests and no significant sex interaction.

The non-linear relationships (in the overall sample with the p for non-linearity) are presented in Fig 2. Non-significant curvilinear relationships were observed for the association of daily sitting time with neck, shoulder, and low back pain, whereas the sitting time/ knee pain relationship was observed to be linear. For the subgroup analysis by GMS [results provided in Supplementary S2a–S2d Fig in S1 File], curvilinear relationships were observed in the NGM, prediabetes and T2D groups for all the MSP outcomes but were statistically non-significant, except for knee pain in the prediabetes group which showed a marginally significant non-linear relationship (p for non-linearity = 0.05).

## Discussion

This study uniquely examined the cross-sectional associations of device-derived daily sitting time with MSP in different body regions, including neck, shoulder, low back, and knee pain in middle-aged and older adults, according to their GMS. We found evidence of a significant association of longer hours of daily sitting with higher odds of knee pain in a linear function after adjusting for relevant confounders including BMI, MVPA, and CVD; this remained after accounting for mobility limitations. The association was statistically significant only in those with T2D and not in the prediabetes or NGM groups. No significant associations were observed between daily sitting time and neck, shoulder, or low back pain in the overall sample or the analysis according to GMS–the NGM, prediabetes, or T2D group, as well as statistically non-significant non-linear relationships.

There is the potential for reverse causality bias within the type of cross-sectional analyses undertaken in our study. In this context, MSP could adversely impact physical function and mobility, especially in older adults [30, 31]. Chronic pain syndromes, for instance, are associated with several psychosocial factors which are often characterised by fear about using affected joints [31]. This may result in progressive loss of physical functioning, impaired mobility, limited physical activity behaviours and excessive leisure-time sitting. Alternatively, MSP in older adults, especially in those with T2D, may contribute, in part, to high volumes of daily sitting time [12, 32]. For example, low back or knee pain that is secondary to T2D complications may plausibly lead to mobility limitations and subsequently, more time spent sitting. After excluding those participants with self-reported mobility limitation (about 25.9% of the total analysed sample; see Supplementary S2 Table in S1 File) from our analysis, the observed associations between daily sitting time and knee pain became non-significant, possibly reflecting loss of power, yet the observed trend remained unchanged. Furthermore, the prevalence of large amounts of time spent sitting is high in older adults, particularly so in those with chronic diseases, implying the potential for reverse causation [2, 12, 32]. There is evidence that suggests probable bidirectional associations between pain-related chronic conditions and higher volumes of sitting time [33].

This is one of the first studies to separately report on the associations of daily sitting time with MSP in those with and without T2D. Among the MSP outcomes investigated, we observed a higher prevalence of knee pain in the T2D group than in the prediabetes and NGM groups. Interestingly, a statistically-significant positive association with daily sitting time was observed only in those with T2D, which is assumed to be linearly related. While this finding may be biologically plausible, the statistically non-significant interaction of daily sitting with GMS in our analysis (Fig 1) limits the interpretation of this finding as indicating a significant difference in the association of daily sitting time with knee pain between those with T2D and prediabetes or NGM. The lack of a significant interaction may be due to several factors, including the wide variations in the sample sizes of the NGM, prediabetes, and T2D groups. Nevertheless, we observed that sitting time and knee pain may be non-linearly related in the prediabetes group (Supplementary S2d Fig in S1 File). The evidence on the association between T2D and MSP-related conditions such as knee osteoarthritis has been documented [7, 10]. For example, evidence from a meta-analysis indicates that the odds of incidence and progression of osteoarthritis (mostly of the knee) are higher in those with T2D [7]. This evidence is supported by the findings in the placebo arm of a randomised controlled trial in patients with osteoarthritis, in which the presence of T2D increased the risk of progressive knee joint narrowing [10]. To date, no study has documented the association between sitting time and knee pain in those with T2D. Nevertheless, a population-based study of Korean adults over 50 years has documented a positive cross-sectional association of self-reported daily sedentary behaviour (sitting time) above 10hrs/day with knee pain [34].

The mechanisms underlying MSP conditions in T2D are not well understood; however, they likely involve a complex set of factors associated with T2D, including older age, obesity, and the systemic effect of persistent hyperglycaemia [35, 36]. For instance, mechanisms of knee osteoarthritis in T2D [36] may include biomechanical joint load and systemic inflammatory pathways related to older age and obesity along with those related to hyperglycaemia, including advanced glycation end products (AGEs) and their receptor (RAGE) interaction pathway, as well as reactive oxygen species (ROS) pathway which enhances secretion of pro-inflammatory factors. Collectively, these may contribute to oxidative stress and inflammation processes that promote vascular endothelial dysfunction and joint cartilage degradation [36–38]. In this context, it is relevant to note that our statistical models controlled for BMI. Behavioural factors, including sedentary behaviour may in part contribute to, or augment, some of these potential mechanisms through some of the known cardiometabolic consequences of time spent sitting [39, 40].

There is some supporting evidence from acute experimental studies [39, 41] and observational studies [40, 42] that sedentary time may be unfavourably associated with cardiometabolic biomarkers such as dyslipidaemia, hyperglycaemia, insulin resistance, and vascular endothelial dysfunctions in T2D. Also, an association between higher volumes of sedentary time and unfavourable levels of systemic inflammatory biomarkers in adults living with T2D has been observed [43, 44]. Thus, sedentary behaviour may potentially have some links to the plausible biological pathways of T2D/MSP associations. This may be possible through the influence of sedentary behaviour on insulin resistance, hyperglycaemia, and dyslipidaemia mediating inflammatory changes and impaired blood flow in joints leading to articular surface cartilage degradation [36–38]. In support of this, an epidemiological cross-sectional study observed that an increased prevalence of low back pain in people with T2D was also associated with self-reported sedentary behaviour [6]. Furthermore, there is evidence from a prospective study that higher volumes of sedentary behaviour are associated with increased severity of bodily pain, which is significantly more apparent in people living with T2D [18]. Our observed cross-sectional association of daily sitting time with knee pain in those with T2D after accounting for the confounding bias of BMI, MVPA, and CVD may also support the notion that cardiometabolic and systemic inflammatory effects of sedentary behaviour, which is more pronounced in people with T2D [45, 46], may, in part, play some role in the pathogenesis of knee pain in T2D. However, with our relatively small effect size cross-sectional finding, potential residual confounding effects and reverse causation could be also likely.

We did not observe significant associations between daily sitting time and neck, shoulder, or low back pain in any of the GMS groups. There is an indication that the relationship between sitting time and neck, shoulder, or low back pain may not necessarily be linear but rather curvilinear; however, the observed curvilinear relationships in our study were statistically non-significant (Fig 2). Studies are yet to specifically investigate the associations of daily sitting time/ sedentary behaviour with MSP separately in people with T2D, prediabetes, or NGM, making direct comparison challenging. Previous evidence on these associations, mostly from heterogeneous populations and for diverse sedentary behaviour domains, has been inconsistent [15, 16, 47]. Studies have documented inconsistent evidence on associations of sitting time/ sedentary behaviour with MSP-related outcomes, including neck/shoulder, or low back pain [48–51]. Our findings are consistent with those of a prospective analysis of the Danish Health Examination Survey Cohort 2007–2008 data that showed that self-reported daily sitting time of 10hrs/day or more was not associated with low back pain [51]. In contrast, some Danish studies of tradespeople have reported positive cross-sectional associations of Actigraph-derived daily sedentary time with low back pain [48] and neck/shoulder pain intensity [50]. Similarly, a study of Korean adults aged over 50 years found cross-sectional evidence that self-reported daily sitting time of more than 7hrs/day was associated with low back pain [49].

Several factors may account for the differences between our findings and those of others. Notably, differences in the instruments used to estimate daily sitting/ sedentary time are evident. Body-worn devices provide greater accuracy for estimating sitting time, specifically, the thigh-worn activPAL device used in our study is known to have higher accuracy than the Actigraph device (which primarily detects sitting time) [52, 53]. Self-report measurement instruments, on the other hand, are based on subjective estimates of sitting time or sedentary behaviours and are prone to higher levels of bias [52–54]. The inconsistencies in the evidence may also reflect that the mechanisms that underpin MSP may be complex and differ with respect to the body part involved. Also, heterogeneity in the MSP assessment (acute or chronic pain) among these studies may partly explain the differences.

### Strengths and limitations

The strengths of our study include using the activPAL device to measure daily sitting time, the gold standard research instrument for accurately assessing sitting or lying postures [52, 54], and the large sample size with a substantial number of participants with T2D, which allowed stratified analyses according to GMS. Further, we examined the association in different MSP outcomes, providing the opportunity to compare the associations of daily sitting time with different MSP outcomes by GMS in the same dataset.

Study limitations include the cross-sectional design which precludes causal inference, and as previously referred to, there is also the potential for reverse causation among the observed associations. Furthermore, the participants’ mean daily sitting time was derived from one-week wear of the activPAL data, and participants were included in the analysis if there was at least one valid day of device-wear time. This may not reflect the studied participants’ true habitual daily sitting behaviour. In addition, aside from the confounders for which we adjusted, there may be other unmeasured confounders, such as occupational physical activity behaviours which were not accounted for in the analyses. Also, there is no universally accepted measure of musculoskeletal pain for epidemiological studies. The MSP assessment tool used in our study has limitations inherent to self-report instruments, including that the inclusion of data from some “high reporters” of pain may bias the findings [55, 56]. Also, the assessment of acute MSP (at least one instance of experiencing pain for the past one month) might be too sensitive, with lower specificity to effectively discriminate MSP among the participants, thereby masking the potential associations.

### Implications for research and practice

Our findings may provide new insights for future research and clinical implications. The primary focus of this study was to better understand the associations of total volumes of daily sitting time with MSP; however, it is well recognised that sitting time is accumulated across multiple domains (at home, work, leisure, or commuting in a vehicle) which could be of public health interest [57]. For instance, recent evidence suggests that the associations of domain-specific sitting time (e.g., time spent sitting in a car or at a workstation desk) with adverse health outcomes may be more important than just the total volume of sitting/ sedentary time accumulated during the whole day [58, 59]. Moreover, there is evidence that indicates that the association between sitting time and MSP may be influenced by factors of occupational environment structures [13]. For example, high sitting time in tradespeople who engage in labour-intensive work may be inversely associated with neck and low back pain [15], whereas it may be associated with more neck/shoulder and low back pain in office-based workers [13]. Studies have also reported differences in the associations of leisure-time and occupational sitting time with MSP, as well as the pattern of accumulation of the sitting time with MSP [17, 48].

Future studies, preferably utilising prospective designs could focus on investigating the associations of domain-specific sitting time and the pattern of sitting (sitting bouts) with MSP according to GMS in different occupational groups. The association of daily sitting time with knee pain could be explored by examining the association of sitting bout duration with knee pain to better understand sitting patterns that are more likely to be adversely associated with knee pain, especially in those living with T2D. Also, the composition of daily sitting time relative to time spent stepping and standing in relation to MSP-related conditions, particularly with knee pain according to GMS and potential associated mobility limitations could be examined in future studies. Furthermore, studies could examine MSP-related conditions as exposures that may influence sitting behaviour outcomes, as well as the potential interaction role of GMS in such relationships.

Notwithstanding the potential for reverse causality, these findings suggest that some MSP conditions, specifically knee pain, may also be added to the numerous adverse health outcomes that have been shown to be detrimentally associated with higher volumes of sitting time [2, 60]. Accumulated evidence indicates that interrupting prolonged sitting time with, at least, light-intensity physical activity breaks such as standing or light-walking may induce health benefits [61, 62]. This has prompted new recommendations to replace sedentary time with physical activity of any intensity within the 2020 World Health Organisation physical activity and sedentary behaviour guidelines [63], and within the American Diabetes Association guidelines to specifically improve glycaemic management in people with T2D and prevent T2D in those at risk [64]. Our findings suggest that there may be further benefits for people living with T2D, especially middle-aged and older adults with coexisting MSP-related conditions [65, 66].

## Conclusion

In this study, we observed that device-assessed daily sitting time was associated with higher odds of knee pain in middle-aged and older adults with the association being most evident in those with T2D. There were no significant associations with neck, shoulder, or low back pain. The non-linear relationships of sitting time with the MSP outcomes were statistically non-significant. Further studies, using prospective study designs, should focus on examining the potential associations (linear and non-linear) of domain-specific sitting time (including leisure time, work, and transport) and of sitting bout patterns with knee pain and other MSP-related conditions according to GMS. This will help better understand whether particular thresholds of daily sitting time are associated with an increased risk of future knee pain, as a basis for future intervention trials to reduce time spent sitting, particularly in the context of mobility limitations for those with T2D.

## Supporting information

S1 File (PDF)
