# Supplementary material for: Device-measured sitting time and musculoskeletal pain in adults with normal glucose metabolism, prediabetes and type 2 diabetes–The Maastricht Study
Source: PLoS One. 2023 May 4;18(5):e0285276. doi: 10.1371/journal.pone.0285276 (PMC10159126; doi:10.1371/journal.pone.0285276)
Supplement: S1 File — (PDF) [file pone.0285276.s001.pdf]

Supplementary File: Device-measured sitting time and musculoskeletal pain in adults with normal glucose metabolism, prediabetes and type 2 diabetes – the Maastricht Study

Tables

Supplementary Table S1: Association between the interaction of daily sitting time (hours/day) with glucose metabolism status (GMS) and musculoskeletal pain (MSP) outcomes

MSP outcomes	Daily sitting time# GMS model	
	OR (95%CI)	p-value
**Neck pain**		
Daily sitting time	1.00 (0.93 – 1.06)	0.888
NGM	Reference	
Prediabetes	0.93 (0.29 – 2.93)	0.895
T2D	0.92 (0.32 – 2.61)	0.872
Interaction terms		
Daily sitting time#NGM	Reference	
Daily sitting time#Prediabetes	0.99 (0.88 – 1.12)	0.926
Daily sitting time#T2D	0.99 (0.89 – 1.10)	0.819
**Shoulder pain**		
Daily sitting time	0.99 (0.92 – 1.05)	0.679
NGM	Reference	
Prediabetes	1.04 (0.32 – 3.39)	0.945
T2D	0.63 (0.22 – 1.81)	0.388
Interaction terms		
Daily sitting time#NGM	Reference	
Daily sitting time#Prediabetes	1.01 (0.89 – 1.14)	0.903
Daily sitting time#T2D	1.07 (0.96 – 1.19)	0.241
**Low back pain**		
Daily sitting time	1.00 (0.94 – 1.06)	0.980
NGM	Reference	
Prediabetes	0.52 (0.17 – 1.64)	0.265
T2D	0.80 (0.29 – 2.24)	0.674
Interaction terms		
Daily sitting time#NGM	Reference	
Daily sitting time#Prediabetes	1.08 (0.94 – 1.22)	0.224
Daily sitting time#T2D	0.80 (0.92 – 1.14)	0.682
**Knee pain**		
Daily sitting time	1.05 (0.99 – 1.13)	0.119
NGM	Reference	
Prediabetes	1.42 (0.42 – 4.78)	0.567
T2D	0.70 (0.24 – 2.08)	0.523
Interaction terms		
Daily sitting time#NGM	Reference	
Daily sitting time#Prediabetes	0.97 (0.85 – 1.10)	0.623
Daily sitting time#T2D	1.06 (0.95 – 1.18)	0.312

MSP: Musculoskeletal pain | NGM: Normal Glucose Metabolism | T2D: Type 2 Diabetes | OR: Odds ratio | CI: Confidence Interval

Note: The model Adjusted for age and sex

Supplementary Table S2: Sensitivity analysis to check the associations after excluding the 25.9% of participants with mobility limitations
[daily sitting time (hours/day) and musculoskeletal pain (MSP) outcomes]

MSP outcomes	N	Model A	Model B	Model C
		OR (95%CI)	OR (95%CI)	OR (95%CI)
**Neck pain**				
Overall	2,095	1.01 (0.95 – 1.06)	1.00 (0.94 – 1.07)	1.02 (0.96 – 1.08)
NGM	1,410	1.02 (0.95 – 1.09)	1.00 (0.93 – 1.08)	1.03 (0.95 – 1.11)
Prediabetes	305	0.97 (0.85 – 1.11)	0.98 (0.84 – 1.14)	0.98 (0.84 – 1.14)
T2D	380	1.04 (0.92 – 1.18)	1.06 (0.92 – 1.21)	1.06 (0.92 – 1.21)
**Shoulder pain**				
Overall	2,095	1.01 (0.95 – 1.07)	0.99 (0.93 – 1.05)	1.00 (0.93 – 1.06)
NGM	1,410	0.99 (0.92 – 1.07)	0.97 (0.90 – 1.05)	0.98 (0.91 – 1.07)
Prediabetes	305	1.05 (0.91 – 1.20)	1.02 (0.87 – 1.19)	1.01 (0.86 – 1.18)
T2D	380	1.02 (0.90 – 1.16)	1.00 (0.87 – 1.15)	0.99 (0.86 – 1.15)
**Low back pain**				
Overall	2,095	1.01 (0.96 – 1.07)	1.01 (0.95 – 1.08)	1.03 (0.97 – 1.09)
NGM	1,410	1.01 (0.94 – 1.08)	1.02 (0.95 – 1.10)	1.03 (0.95 – 1.11)
Prediabetes	305	1.12 (0.98 – 1.28)	1.12 (0.96 – 1.30)	1.14 (0.98 – 1.34)
T2D	380	0.95 (0.84 – 1.08)	0.92 (0.80 – 1.05)	0.91 (0.80 – 1.05)
**Knee pain**				
Overall	2,095	1.04 (0.98 – 1.11)	1.04 (0.98 – 1.11)	1.04 (0.97 – 1.11)
NGM	1,410	1.04 (0.96 – 1.13)	1.04 (0.96 – 1.13)	1.03 (0.95 – 1.12)
Prediabetes	305	1.00 (0.86 – 1.16)	1.02 (0.86 – 1.20)	1.00 (0.84 – 1.19)
T2D	380	1.06 (0.93 – 1.21)	1.08 (0.94 – 1.25)	1.09 (0.95 – 1.26)

MSP: Musculoskeletal pain | N: Sample size | NGM: Normal Glucose Metabolism | T2D: Type 2 Diabetes | OR: Odds ratio | CI: Confidence Interval
Model A: Adjusting for age and sex. **Model B:** Adjusting for covariates in Model A + BMI and MVPA. **Model C:** Adjusting for covariates in Model B + Education level, employment status, smoking status, DHD-index, and history of cardiovascular disease.

Supplementary Table S3: Models' fitness checks

Models	Pseudo R^2	AIC	BIC	Log likelihood	Likelihood ratio test p-value
Neck pain					
Linear model	0.0268	3834.134	3923.339	-1902.067	0.5914
Non-linear model (RCS)	0.0268	3835.846	3931.997	-1901.9229	
Shoulder pain					
Linear model	0.0150	3716.237	3805.441	-1843.1183	0.8394
Non-linear model (RCS)	0.0150	3718.195	3813.347	-1843.0977	
Low back pain					
Linear model	0.0088	3905.441	3994.646	-1937.7205	0.9155
Non-linear model (RCS)	0.0088	3907.43	4002.581	-1937.7149	
Knee pain					
Linear model	0.0275	3561.082	3650.287	-1765.541	0.9957
Non-linear model (RCS)	0.0275	3563.082	3658.233	-1765.541	

Supplementary Table S4: Restricted cubic spline knots selection

No. of knots	AIC	BIC
3 knots	3835.846	3930.997
4 knots	3836.69	3937.789
5 knots	3840.297	3965.183

Figures

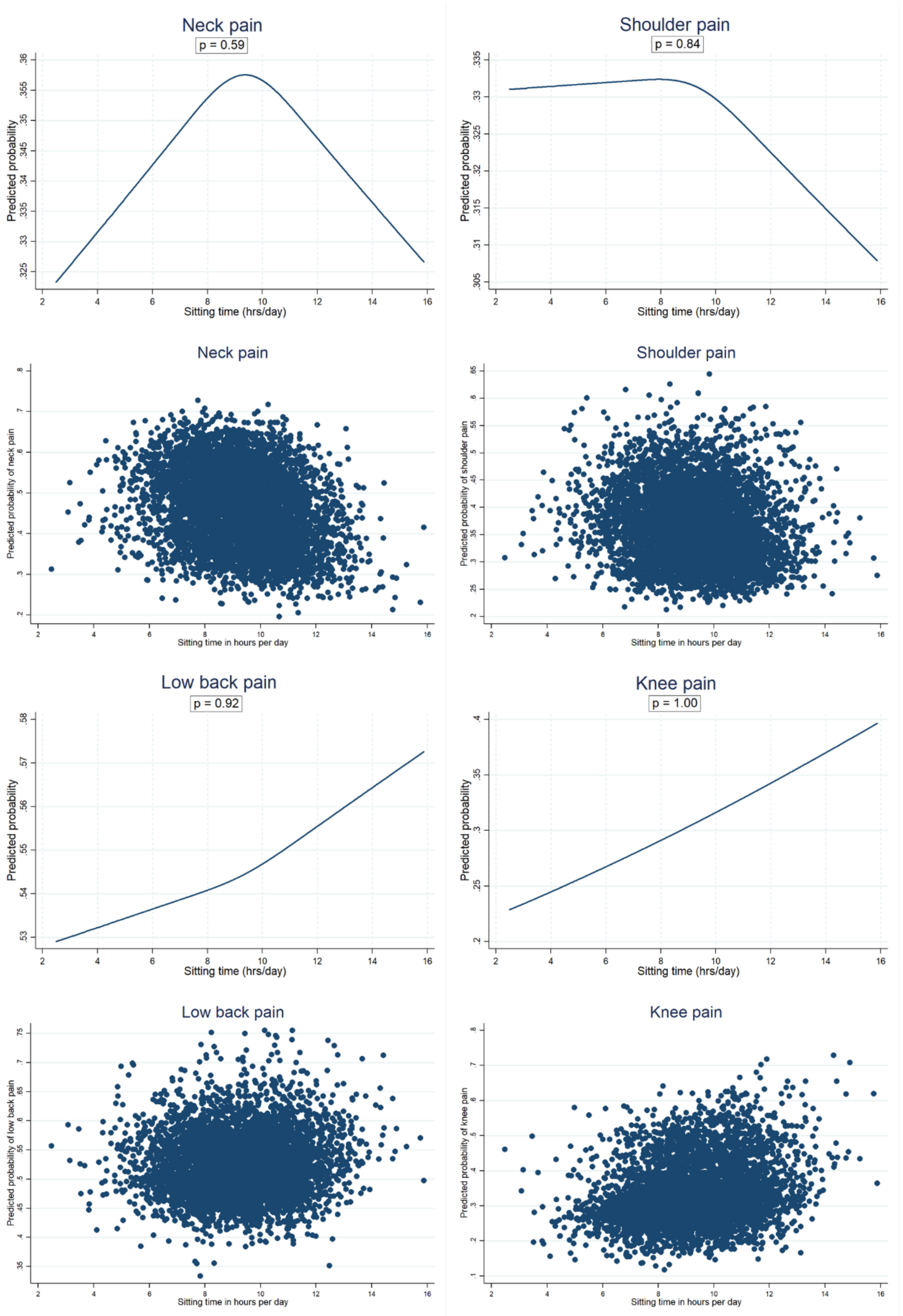

Supplementary Figure S1: Non-linear relationships between daily sitting time and neck, shoulder, low back, and knee pain, as well as the scatter plots of the predicted probability of the musculoskeletal pain outcomes with sitting time in the overall sample.

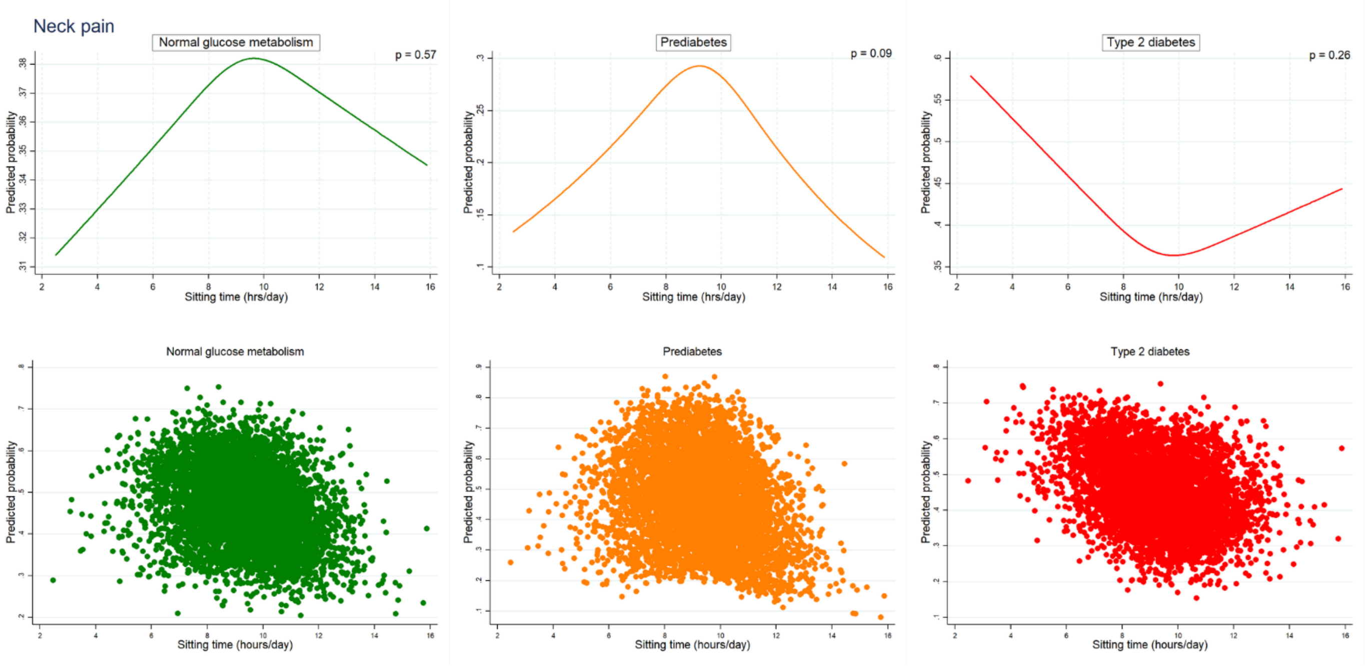

Supplementary Figure S2a: Non-linear relationships between daily sitting time and neck pain, as well as the scatter plots of the predicted probability of neck pain with sitting time in those with normal glucose metabolism, prediabetes, and type 2 diabetes.

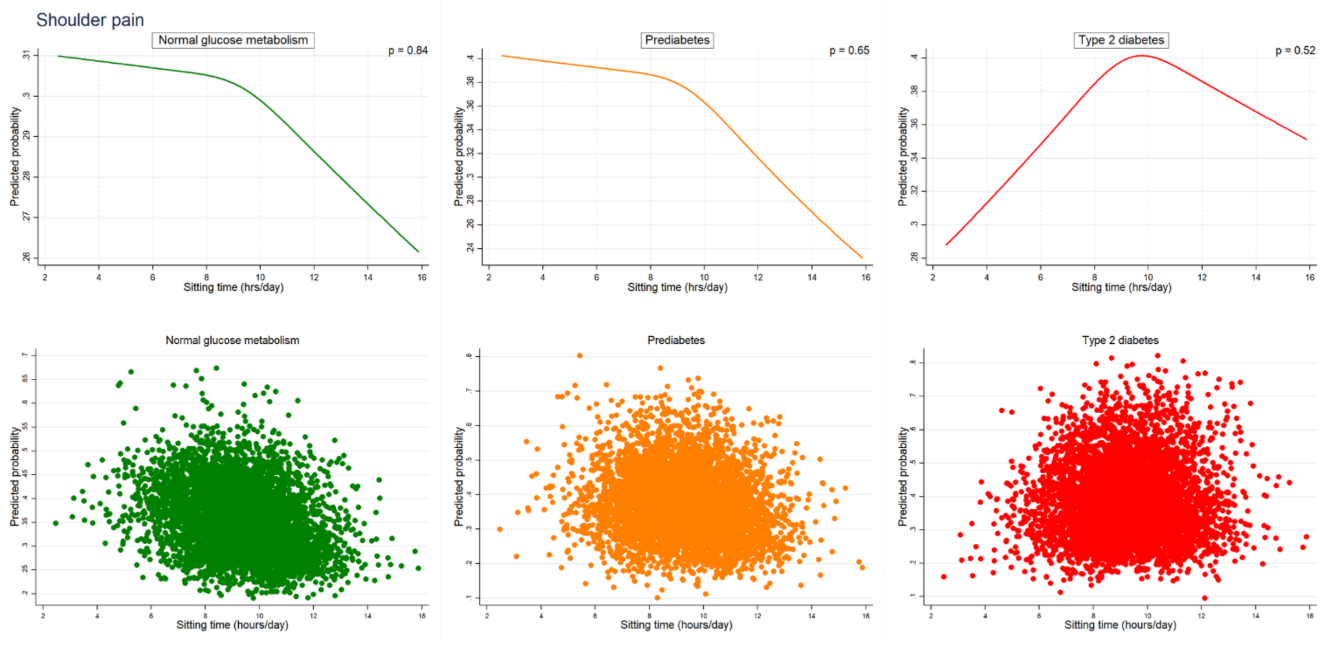

Supplementary Figure S2b: Non-linear relationships between daily sitting time and shoulder pain, as well as the scatter plots of the predicted probability of shoulder pain with sitting time in those with normal glucose metabolism, prediabetes, and type 2 diabetes.

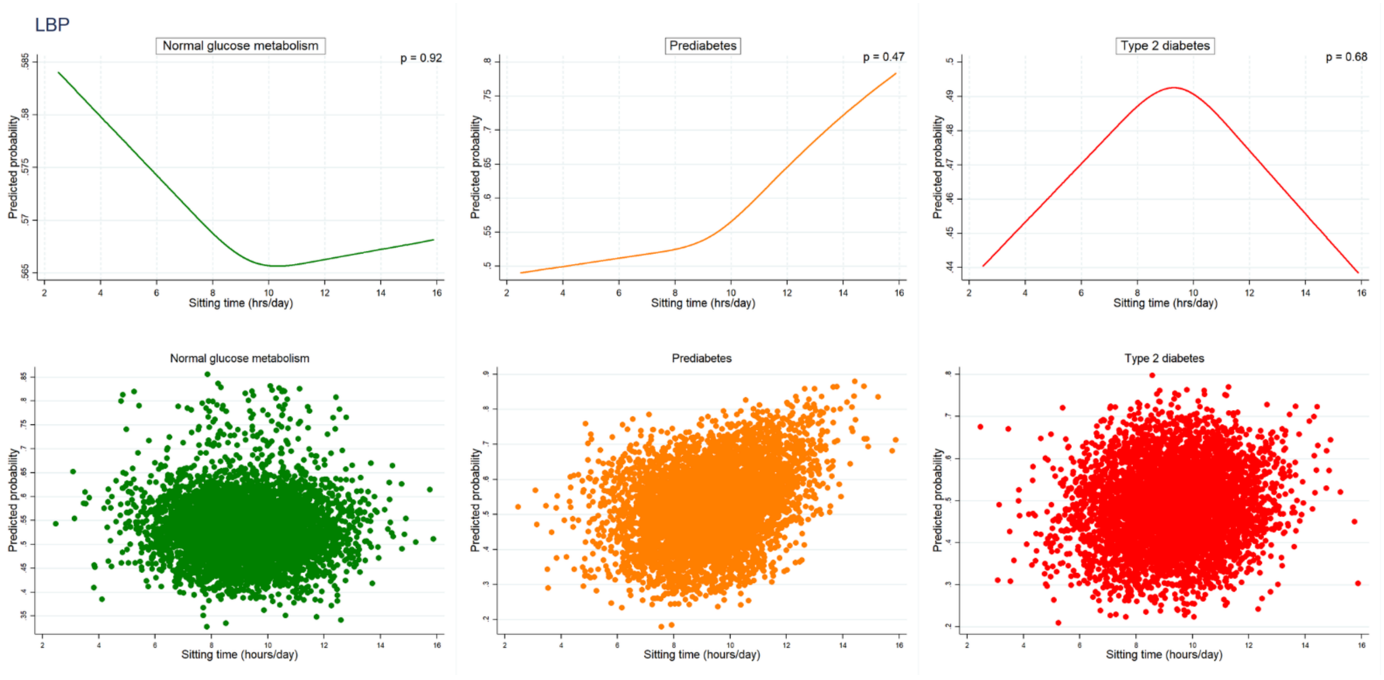

Supplementary Figure S2c: Non-linear relationships between daily sitting time and low back pain (LBP), as well as the scatter plots of the predicted probability of low back pain (LBP) pain with sitting time in those with normal glucose metabolism, prediabetes, and type 2 diabetes.

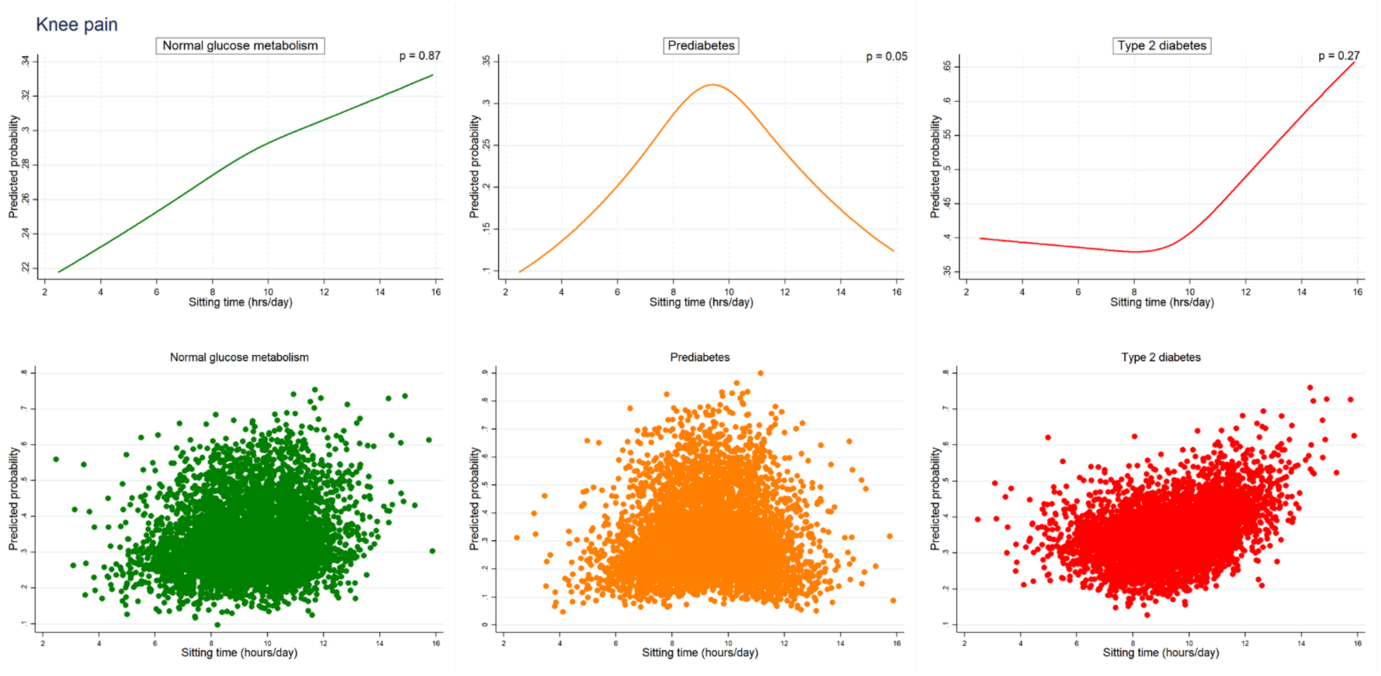

Supplementary Figure S2d: Non-linear relationships between daily sitting time and knee pain, as well as the scatter plots of the predicted probability of knee pain with sitting time in those with normal glucose metabolism, prediabetes, and type 2 diabetes.

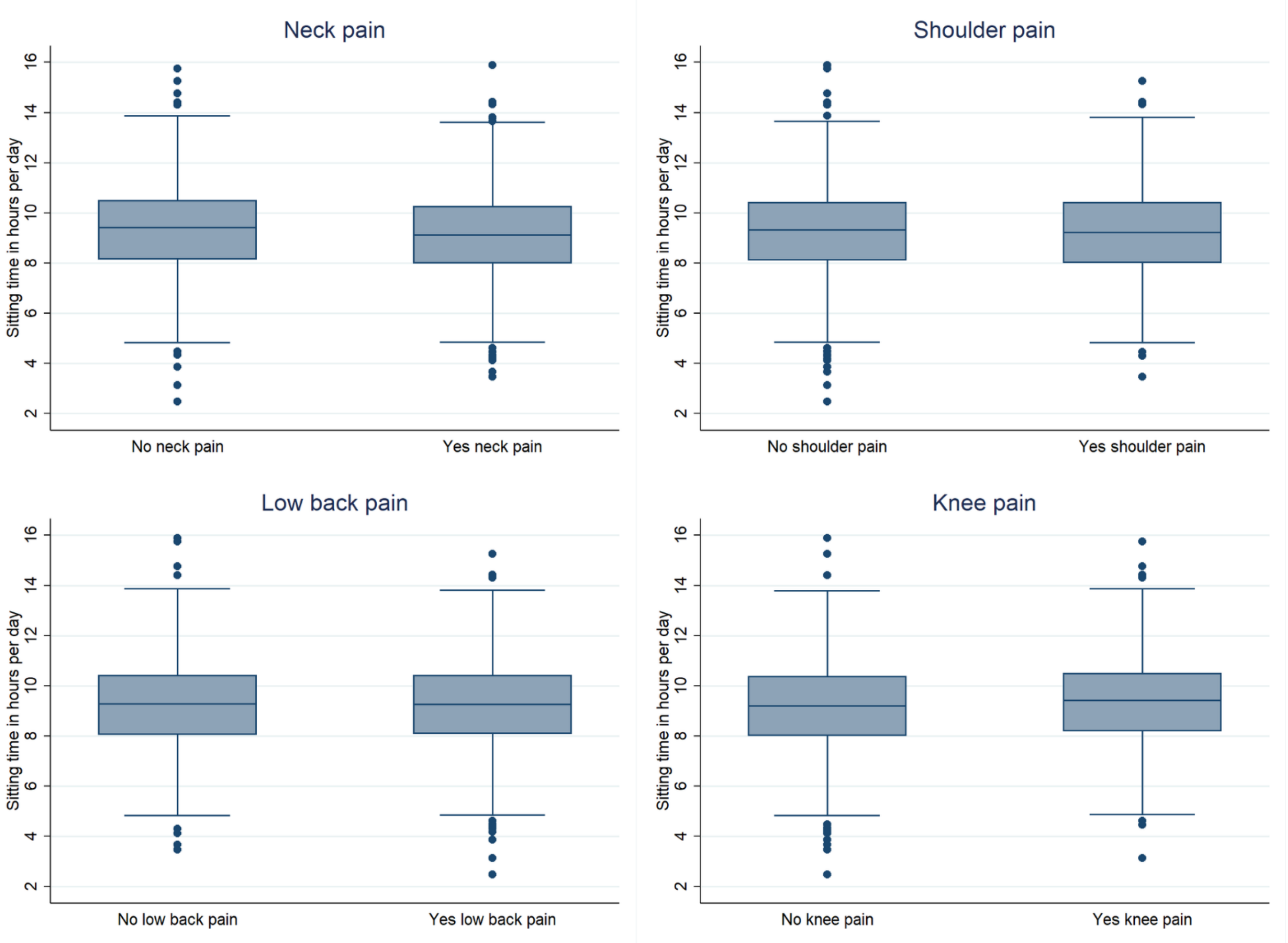

Supplementary Figure S3: Distribution of daily sitting time by musculoskeletal pain outcomes

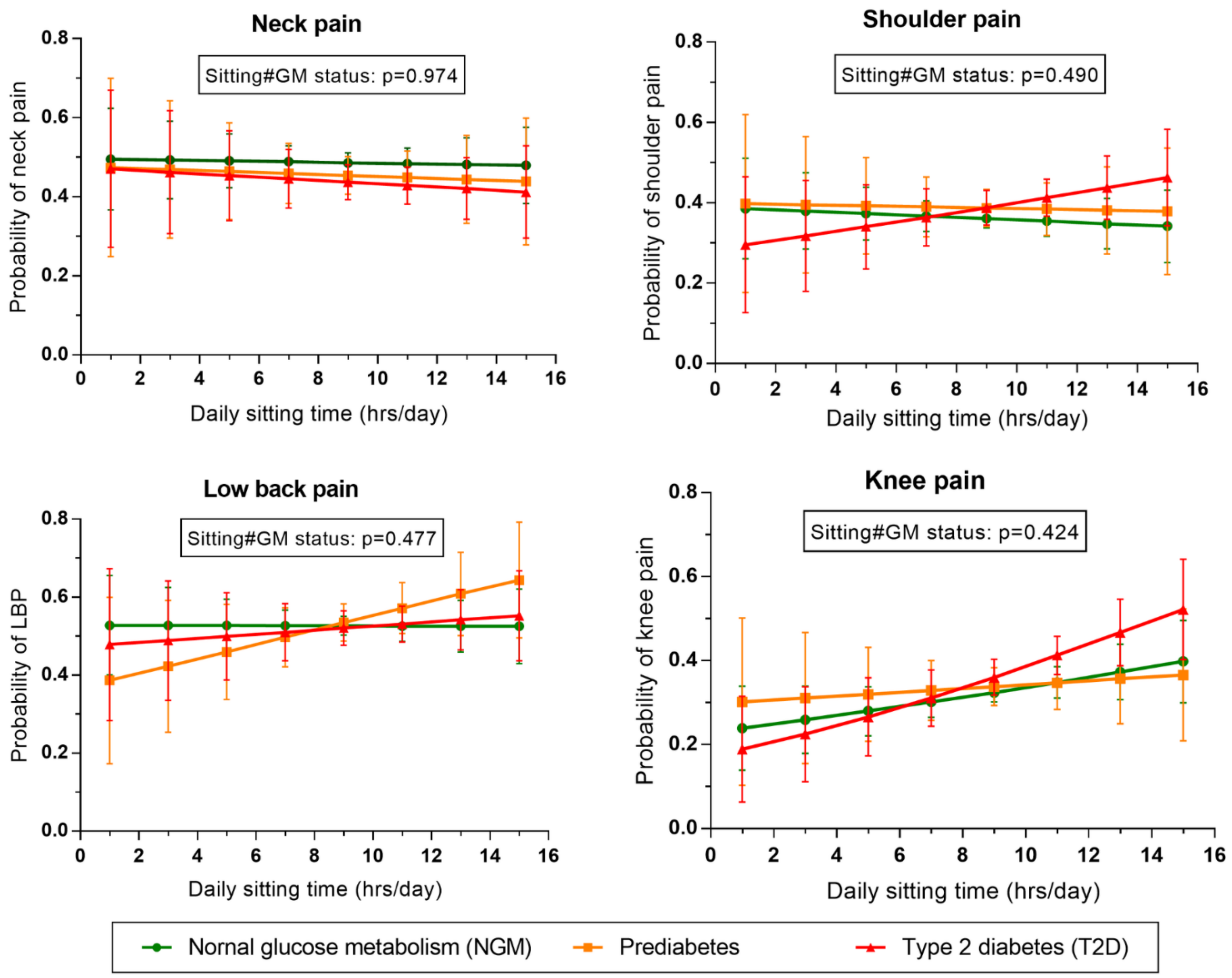

Supplementary Figure S4: The predictive probability of the musculoskeletal pain outcomes with daily sitting time according to glucose metabolism status (GMS).

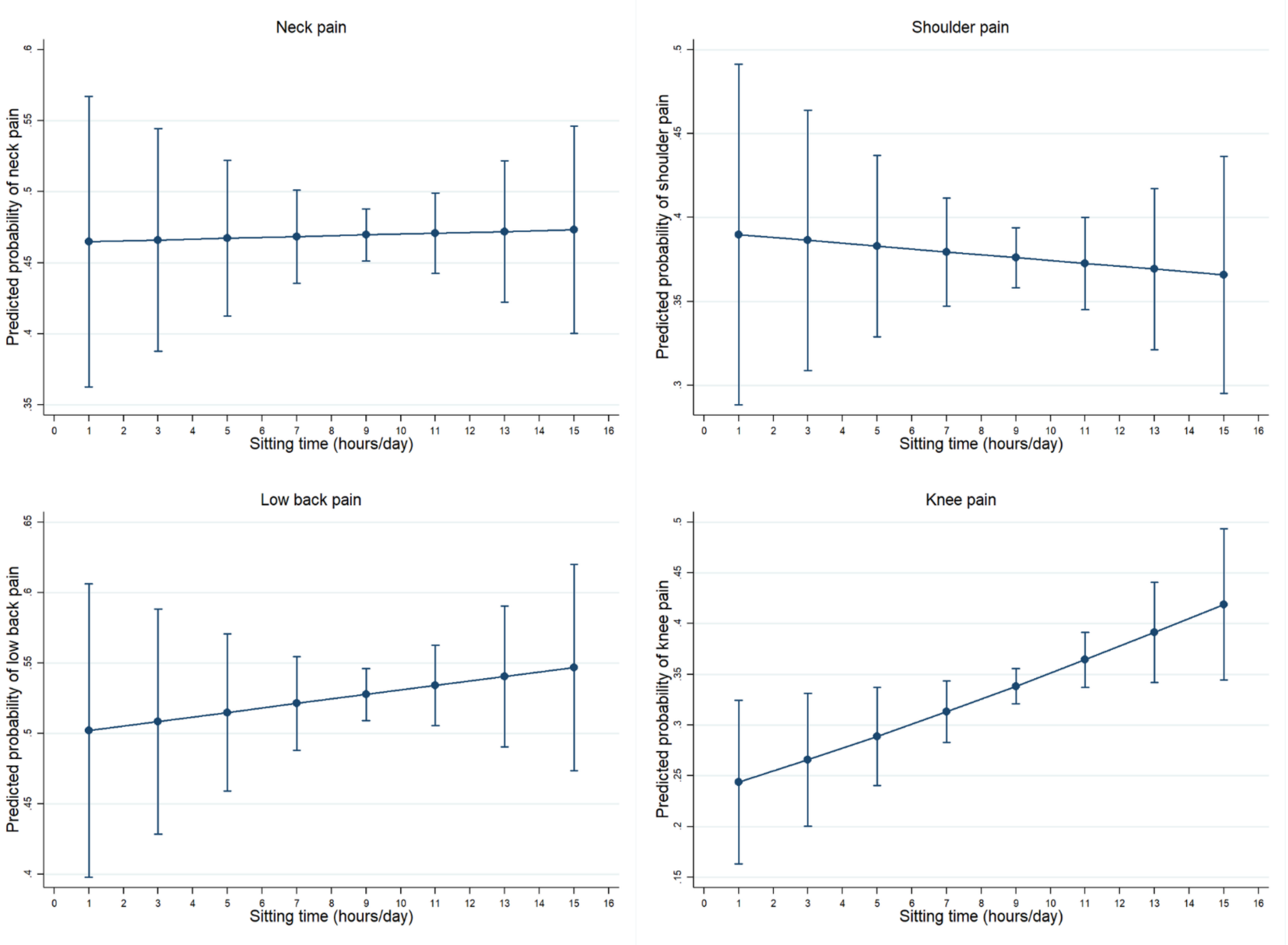

Supplementary Figure S5: Plots of predicted probability of the musculoskeletal pain outcomes with daily sitting time
